# Development of tenecteplase for stroke thrombolysis: Japan’s endeavor

**DOI:** 10.1038/s41440-025-02404-8

**Published:** 2025-10-24

**Authors:** Kazunori Toyoda

**Affiliations:** Department of Cerebrovascular Medicine, National Cerebral and Cardiovascular Center, Suita, Japan

**Keywords:** Asia, hypertension, Ischemic stroke, Reperfusion therapy, Tissue-type plasminogen activator

## Abstract

Intravenous thrombolysis for acute ischemic stroke is strongly recommended based on high-level evidence. However, the efficacy of the currently approved drug, alteplase, is not necessarily sufficient. Thrombolysis can trigger intracranial hemorrhage, that is a critical issue that cannot be overlooked, especially for Asians, who are generally hypertensive and prone to bleeding, making strict blood pressure control essential. Based on clinical trial results of a new thrombolytic agent, tenecteplase, several guidelines now recommend its use, and the off-label use has become common in many countries. As of 2024, regulatory approval has been granted in Western countries. Approval is also expected in major Asian countries between 2025 and 2026. In Japan, approval requires clinical data specific to Japanese patients, but no domestic pharmaceutical company currently handles the drug. We are conducting an investigator-initiated, multicenter, prospective, randomized, open-label, masked-endpoint, superiority trial, T-FLAVOR, comparing tenecteplase at 0.25 mg/kg (international standard dose) with alteplase at 0.6 mg/kg (a unique low dose approved in Japan). The target population consists of patients with ischemic stroke due to large vessel occlusion within 4.5 h of onset, who are also eligible for mechanical thrombectomy within 6 h. The primary efficacy outcome is successful early reperfusion assessed via catheter angiography after drug administration. Enrollment of the planned 220 participants was complete, and results are expected to be published in 2025. This is the only clinical trial using tenecteplase in Japan and the world’s first to directly compare tenecteplase and low-dose alteplase (0.6 mg/kg).

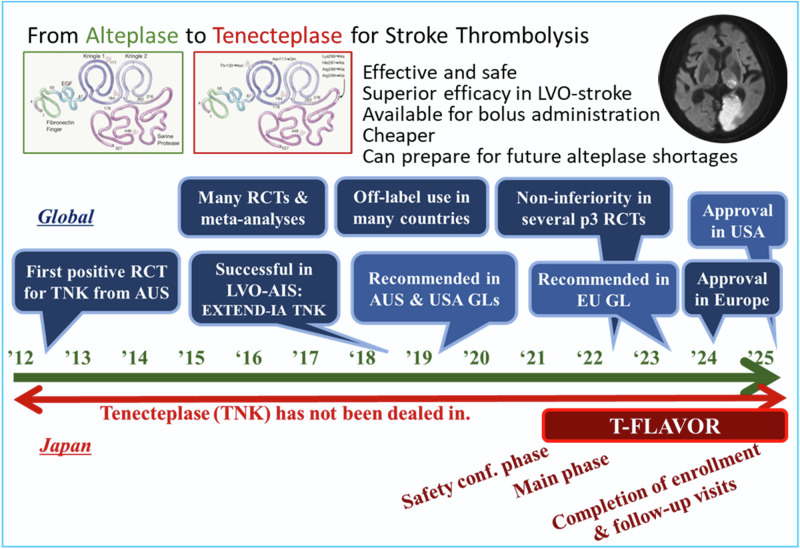

## Introduction

Stroke is a major public health concern in East Asia, including Japan, where it ranks as the fourth leading cause of death and the second most common cause of long-term disability requiring care. According to information collected between 2000 and 2019 from the Japan Stroke Data Bank, an ongoing, hospital-based, multicenter, prospective registry of hospitalized patients with acute stroke based on a web database, ischemic stroke accounted for 74% of overall patients with acute stroke [1].

Intravenous thrombolysis and mechanical thrombectomy are both standard treatments in the hyperacute phase of ischemic stroke recommended in global stroke guidelines with a high level of evidence. These therapies aim to rapidly recanalize occluded cerebral arteries, rescue the ischemic penumbra surrounding the ischemic core, and thereby improve outcomes. Among these, intravenous thrombolysis is applicable across various subtypes of ischemic stroke. In the Japan Stroke Data Bank, neurological severity at admission shown by the National Institutes of Health Stroke Scale (ranging from 0 to 42, with higher score indicating more severe stroke) score was 12 in median for thrombolyzed patients relative to 4 for the other ischemic stroke patients. At discharge, 43% of thrombolyzed patients achieved functional independence (modified Rankin Scale [mRS] 0–2; the scale ranges from 0 [no symptoms] to 6 [death] [1, 2]. Intravenous thrombolysis is available 24/7 in nearly 1000 primary stroke centers throughout Japan. However, intracranial hemorrhage after thrombolytic administration is an especially critical issue that cannot be overlooked. Especially, Asians are generally hypertensive and prone to intracranial hemorrhage that is reportedly 2–4 times more common in Asian than in Caucasians [3–5]. Thus, strict blood pressure control is essential for Asians.

The thrombolytic agent used for ischemic stroke has long been limited to alteplase, a recombinant tissue-type plasminogen activator (rt-PA). While many clinical trials attempted to apply other thrombolytic agents, such as prourokinase and desmoteplase, none succeeded in obtaining regulatory approval, either because the agents were not advanced to a larger-scale development trial or because it failed to demonstrate favorable results in such a trial [6, 7]. Against this backdrop, tenecteplase has been under active development internationally for stroke treatment [8].

This article reviews the past, present, and future prospects of thrombolytic therapy for ischemic stroke both within and outside Japan, especially using the promising tenecteplase. In Europe and the United States, the number of clinical development trials for tenecteplase has gradually increased over the course of the past more than 10 years, ultimately culminating in regulatory approval. In East Asia, China has recently published multiple clinical trial results [9–11], and South Korea is in negotiations with regulators for approval. In Japan, the approval of alteplase for use in patients with acute ischemic stroke was considerably delayed, and now, the regulatory process for tenecteplase is likewise encumbered by protracted obstacles. To overcome this critical juncture, an investigator-initiated trial is underway. The trial compares tenecteplase at 0.25 mg/kg (international standard dose) with alteplase at 0.6 mg/kg (a unique low dose approved in Japan [12]) for the first time.

## Development and delay of thrombolytic therapy in Japan

Alteplase was first approved for ischemic stroke in the United States in 1996 [13]. Around the same time, Japan developed a domestic rt-PA, duteplase, which showed favorable results in randomized controlled trials (RCTs) [14, 15]. However, due to a patent-infringement lawsuit, the Japanese company lost the case, and the development of duteplase was halted. Following this setback, momentum grew to introduce alteplase in Japan. In 2002, the Japan Alteplase Clinical Trial (J-ACT), a single-arm open-label trial, was initiated using a lower dose of 0.6 mg/kg compared to the international standard of 0.9 mg/kg [16]. The dose decision was supported by findings from acute myocardial infarction trials, where optimal doses in Japanese patients were lower, ranging from 0.5 to 0.75 mg/kg, than the ~1.25 mg/kg recommended in Western populations and the finding that the optimal dose of duteplase, considered to be 20 million international units, corresponds to 0.57 mg/kg in a 60 kg patient [12]. Concerns about intracranial hemorrhage, more prevalent in the Japanese population, influenced the lower dose selection. J-ACT found that outcomes such as independence at 3 months (mRS 0–1) and early symptomatic intracranial hemorrhage were within acceptable ranges compared to predetermined thresholds based on meta-analyses of foreign trials. Based on these results, 0.6 mg/kg alteplase was approved for use in Japan in 2005. This approval marked a major advancement in stroke emergency care and the establishment of stroke units in Japan. However, it was 9 years later than United States approval, during which stroke care in Japan lagged significantly. Notably, during this period, several prominent public figures, including (ex-)prime ministers and head coaches of the national sport teams, suffered strokes and died or were forced to retire without receiving thrombolytic therapy. Differences in the systems for drug approval and medical insurance within and outside Japan might have been one of the causes of the delay. A typical example is the insurance coverage for off-label use of unapproved drugs, that is highly restricted in Japan but handled more flexibly in some other countries, constituting up to one-third of all prescriptions for common drugs in the United States [17]. An explanation will be provided in a later paragraph taking the development of tenecteplase as an example.

Thrombolytic agents, while effective, carry a risk of bleeding complications; the risk increases even over a short period of time after stroke onset. Initially, alteplase was limited to administration within 3 h of stroke onset globally. In 2008, the European Cooperative Acute Stroke Study (ECASS) III trial in Europe demonstrated the efficacy and safety of treatment between 3 and 4.5 h of onset [18]. In Japan, a petition was submitted by the Japan Stroke Society to the Ministry of Health, Labour and Welfare, and the extension was approved in 2012 without the requirement for an independent confirmatory trial [19].

Despite the extended time window, some patient groups remain ineligible for treatment: such as those who experience strokes during sleep or those with communication impairments and no witnesses at onset. These account for about 20% of all stroke cases. Advanced imaging, including the diffusion-weighted image (DWI: which detects early ischemia within 1 h of onset) and the fluid attenuated inversion recovery image (FLAIR: which requires 3–6 h to visualize changes), allows estimation of stroke onset time by the difference in findings of ischemia between the images [20, 21]. The Thrombolysis for Acute Wake-Up and Unclear-Onset Strokes With Alteplase at 0.6 mg/kg (THAWS) trial in Japan did not demonstrate the efficacy of intravenous thrombolysis in patients selected using DWI-FLAIR mismatch [22], but a pooled analysis with similar international trials (WAKE-UP, EXTEND, and ECASS IV) confirmed its effectiveness, leading to guideline expansion in Japan without significant delay [23].

## Scientific evidence and global developments for tenecteplase

Tenecteplase is a genetically engineered variant of alteplase, where amino acids 296–299 (lysine, histidine, arginine, arginine) are replaced with alanine. It has higher fibrin specificity, a six-fold longer half-life (20–24 min), and greater resistance to plasminogen activator inhibitor-1 (PAI-1) than alteplase, allowing effective thrombolysis with a single bolus injection [24]. In acute ischemic stroke, tenecteplase causes significantly less disruption to the coagulation and fibrinolytic systems than alteplase, consistent with the trend toward reduced incidence of intracranial hemorrhage [25]. In a large multicenter RCT for the approval of tenecteplase for myocardial infarction patients, it was shown not to increase the risk of intracranial hemorrhage compared to alteplase [26]. A meta-analysis of 40 RCTs involving 128,071 patients with myocardial infarction from various countries, demonstrated that tenecteplase was relatively safe in terms of major bleeding among various combinations of antithrombotic agents and thrombolytics, including alteplase [27].

**Fig. 1:**
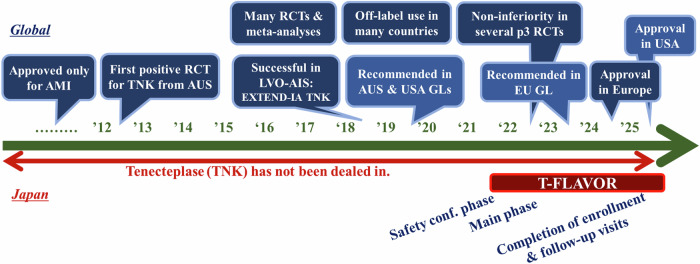
The trend of tenecteplase development. AIS acute ischemic stroke, AMI acute myocardial infarction, AUS Australia, GL guideline, LVO large vessel occlusion, RCT randomized controlled trial, TNK tenecteplase

The first positive stroke trial for tenecteplase was published from Australia in 2012 (Fig. 1) [28]. The phase II trial compared 25 patients in each group receiving alteplase (0.9 mg/kg) or two doses of tenecteplase (0.1 mg/kg and 0.25 mg/kg) and showed a dose-dependent improvement in recanalization and neurological outcomes with tenecteplase. The Tenecteplase versus Alteplase before Endovascular Therapy for Ischemic Stroke (EXTEND-IA TNK) trial in Australia enrolled 202 patients eligible for mechanical thrombectomy within 4.5 h [29]. Tenecteplase (0.25 mg/kg) achieved significantly better early recanalization (22% vs. 10%) and functional outcomes (median mRS: 2 vs. 3) without increasing symptomatic intracranial hemorrhage. Five large phase III RCTs published since 2022 have consistently demonstrated the non-inferiority of tenecteplase (0.25 mg/kg) compared to alteplase (0.9 mg/kg) for achieving complete independence at 3 months (mRS 0–1) in patients treated within 4.5 h of onset (note that non-inferiority was achieved in the per-protocol population, but not in the intention-to-treat analysis in the Tenecteplase versus Alteplase for Stroke Thrombolysis Evaluation [TASTE]) [9, 10, 30–32]. A meta-analysis of 11 such trials showed significantly higher rates of favorable outcomes with tenecteplase, with no increase in symptomatic intracranial hemorrhage or mortality (Table 1) [33]. Meta-analyses planned by European Stroke Organisation found significantly better outcomes with tenecteplase as compared to alteplase in large vessel occlusion, including higher rates of mRS 0–2 (odds ratio [OR] 1.91, 95% confidence interval [CI]: 1.05–3.48) and 0–1 (1.69, 1.15–2.47) at 90 days and improved shift analysis results (1.63, 1.05–2.54) [34]. The Tenecteplase Treatment in Ischemic Stroke involving 588 stroke patients with large vessel occlusion showed over 20% early recanalization after tenecteplase, regardless of time to thrombectomy, suggesting it may reduce the need for mechanical intervention [35].

**Table 1:** Tenecteplase (0.25 mg/kg) vs alteplase (0.9 mg/kg) in stroke within 4.5 h: results from a meta-analysis

Outcome	Risk ratio (95% CI)	*P* Value	*I*^2^; *p* for Cochran *Q*
Excellent functional outcome at 3 months (modified Rankin scale [mRS] 0–1)	1.05 (1.01–1.10)	0.012	0%; 0.85
Good functional outcome at 3 months (mRS 0–2)	1.03 (0.99–1.07)	0.142	28%; 0.18
Reduced disability at 3 months (≥1-point reduction across all mRS strata)	1.10 (1.01–1.19)	0.034	0%; 0.90
Symptomatic intracranial hemorrhage	1.12 (0.83–1.53)	0.456	0%; 0.96
All-cause mortality at 3 months	0.97 (0.82–1.15)	0.727	12%; 0.33

Adapted and cited from reference [33]

Eleven RCTs, including 5 phase III ones (AcT [30], TRACE-2 [9], ATTEST-2 [31], ORIGINAL [10] and TASTE [32]), are studied

These data have led international guidelines to recommend tenecteplase for stroke treatment, despite its unapproved status in many regions [34, 36]. Since tenecteplase had originally been approved as a treatment for myocardial infarction, it was relatively easy for several countries to start its off-label clinical use for stroke patients in late 2010’s based on some promising trials as a theoretical rationale [37]; this would have facilitated the regulatory approval. In 2023, the manufacturer applied for regulatory approval in Europe, and as of 2024–2025, it has been approved in many European countries and the United States. In contrast, tenecteplase itself does not exist and has never used for patients with myocardial infarction in Japan. Importation of tenecteplase became a major obstacle to the progress of a domestic trial as described below. However, even if the drug has existed, the strict restrictions on off-label use and the associated issues of reimbursement likely led to a disparity in their dissemination compared with other countries. This point constitutes an issue that cannot be disregarded in the context of Japan’s drug approval and healthcare system.

## Trends in Asia

In Asia, tenecteplase has been used for myocardial infarction. A Korean study showed higher coronary recanalization rates than alteplase [38], while Chinese one reported no significant difference [39]. In stroke care, India and China have long used biosimilar tenecteplase products. Taiwan participated in a global TASTE trial using the original drug [32]. Recently, China has published several RCTs comparing tenecteplase 0.25 mg/kg with alteplase 0.9 mg/kg in stroke patients within 4.5 h. Both the TRACE-2 (using biosimilar tenecteplase) and ORIGINAL (using original tenecteplase) trials demonstrated the non-inferiority of tenecteplase [9, 10]. Although tenecteplase was officially approved for use in patients with AMI in Korea, the approval was withdrawn in 2024 due to decreased usage. An application for official approval to use tenecteplase for acute ischemic stroke was submitted in August 2024 and is currently under review. Regulators in Korea do not require domestic trials for approval. Approval is expected in major Asian countries between 2025 and 2026.

According to a systematic review and meta-analysis comparing the outcomes of tenecteplase treatment between Asian and Caucasian patients, Asians treated with tenecteplase had a higher rate of complete recanalization of occluded cerebral arteries compared to those treated with alteplase (risk ratio [RR]: 1.91, 95% CI: 1.30–2.80), whereas no significant difference was observed among Caucasians (RR: 0.99, 95% CI: 0.87–1.14, Table 2) [40]. Asians were more likely to attain complete recanalization compared with Caucasians (*P* < 0.01). Nevertheless, early neurological improvement was similarly common in both thrombolytic groups even in Asians, and the proportion of patients achieving an mRS score of 0–2 after tenecteplase compared to alteplase was lower and the mortality after tenecteplase compared to alteplase was higher in Asians than Caucasians. No significant racial differences were observed in the incidence of symptomatic intracranial hemorrhage between the two thrombolytic groups. Although the higher rate of complete recanalization after tenecteplase is a promising finding for Asians, the data should be interpreted with caution partly because much fewer Asian patients were included in the analysis of complete recanalization (analyzed by 446 Asians versus 4,970 Caucasians) as compared to other outcomes (ex. mRS 0–2 was analyzed by 2,453 Asians versus 13,509 Caucasians). The large difference in the patient number indicates much fewer experience of trials on thrombolysis in Asia. As underlying characteristics, Asians showed 10% higher frequency of hypertension than Caucasians (65.2% vs. 54.6%, *P* = 0.11). It is desirable that tenecteplase be rapidly adopted in Asian countries and that its therapeutic efficacy in Asian populations be validated in larger cohorts.

**Table 2:** Asians vs Caucasians for stroke thrombolysis: results from a meta-analysis

Outcome	Studies	Risk ratio (95% confidence interval)	*I*^2^ (%)	95% prediction interval	*P* (for subgroup differences)
Complete recanalization					
Asian	4	1.91 (1.30–2.80)	62	0.82–4.43	<0.01
Caucasian	10	0.99 (0.87–1.14)	78	0.35–4.02	
Early neurological improvement					
Asian	4	1.02 (0.92–1.13)	70	0.33–4.50	0.48
Caucasian	8	1.07 (0.98–1.18)	69	0.61–3.04	
Modified Rankin scale 0–2					
Asian	8	1.00 (0.95–1.05)	58	0.94–1.06	<0.01
Caucasian	10	1.14 (1.10–1.19)	48	0.99–1.35	
Modified Rankin scale 0–1					
Asian	3	1.07 (0.99–1.15)	0	0.94–1.21	0.27
Caucasian	12	1.12 (1.07–1.17)	55	0.87–1.34	
Mortality					
Asian	7	1.18 (0.87–1.62)	21	0.81–1.73	0.01
Caucasian	15	1.10 (1.00–1.22)	68	0.45–2.06	
Mixed	5	0.89 (0.79–1.00)	45	0.60–1.35	
Symptomatic intracranial hemorrhage					
Asian	8	1.06 (0.71–1.56)	0	0.67–1.68	0.20
Caucasian	16	0.83 (0.66–1.04)	45	0.41–2.66	
Mixed	4	0.53 (0.28–1.01)	0	0.13–2.18	
Any intracranial hemorrhage					
Asian	7	0.99 (0.76–1.31)	38	0.46–2.42	0.83
Caucasian	7	1.00 (0.86–1.17)	44	0.83–1.21	
Mixed	3	1.08 (0.88–1.34)	0	0.27–4.31	

Adapted and cited from reference [40]

When considering thrombolysis in Asia, drug dosage plays a critical role. As mentioned above, Japan has approved two-thirds of the globally standard dose of alteplase [12, 15]. Some observational studies published from Asia also reported that low-dose alteplase showed generally comparable effectiveness and somewhat better safety compared to the standard-dose treatment [41–44]. In the Taiwan Thrombolytic Therapy for Acute Ischemic Stroke study, particularly among patients aged 70 years or older, the low-dose group had lower rates of symptomatic intracranial hemorrhage and mortality, and a higher proportion of patients achieved a mRS score of 0–2 at 3 months [45]. In the Enhanced Control of Hypertension and Thrombolysis Stroke Study (ENCHANTED) trial, the first trial to directly compare alteplase doses of 0.9 mg/kg and 0.6 mg/kg, with 63% of enrolled patients being Asian, the low-dose group did not demonstrate non-inferiority in the primary outcome of death or disability (mRS 2–6) at 90 days [46]. However, ordinal analysis of mRS scores showed non-inferiority, and the low-dose group experienced significantly lower rates of symptomatic intracranial hemorrhage and fatal events within 7 days. These findings suggest that the optimal dose of alteplase for Asian populations remains uncertain.

What, then, is the optimal dose of tenecteplase? In a meta-analysis comparing different doses of tenecteplase, the 0.25 mg/kg group showed significantly greater early neurological improvement and favorable 90-day outcomes compared to the alteplase group. A network meta-analysis conducted by the European Stroke Organisation demonstrated significant improvements in early neurological improvement within 24–72 h, mRS 0–1, and mRS 0–2, along with marginally better recanalization rate and significantly lower mortality rates after tenecteplase at 0.25 mg/kg than alteplase (Table 3) [47]. In contrast, the 0.40 mg/kg tenecteplase group did not show any improvement in outcomes, along with higher rate of symptomatic intracranial hemorrhage, compared to the alteplase group. What about doses lower than 0.25 mg/kg? Although the sample sizes were small and statistical significance was not reached, the 0.20 mg/kg tenecteplase group showed a trend toward favorable outcomes, with odds ratios comparable to those of the 0.25 mg/kg group. As described in the following chapter, we are currently aiming for regulatory approval of tenecteplase at a dose of 0.25 mg/kg in Japan. After approval, we would like to participate in an international trial to investigate the efficacy of lower doses of tenecteplase.

**Table 3:** Different dose of tenecteplase vs alteplase for stroke outcomes: results from a network meta-analysis

Outcome	0.10 mg/kg	0.20 mg/kg	0.25 mg/kg	0.32 mg/kg	0.40 mg/kg	0.50 mg/kg
Recanalization	1.88 (0.89–3.97)	–	1.37 (0.99–1.89)	–	1.37 (0.71–2.64)	–
Early neurological improvement	1.38 (0.89–2.14)	1.76 (0.92–3.38)	1.52 (1.13–2.05)	1.20 (0.63–2.51)	1.07 (0.84–1.35)	1.18 (0.28–4.97)
Modified Rankin scale 0–1 at 90 days	0.93 (0.63–1.37)	1.24 (0.57–2.70)	1.19 (1.03–1.37)	1.15 (0.60–2.19)	0.91 (0.73–1.14)	1.74 (0.50–6.01)
Modified Rankin scale 0–2 at 90 days	0.87 (0.50–1.52)	1.33 (0.43–4.10)	1.21 (1.05–1.39)	0.96 (0.47–1.96)	0.79 (0.61–1.02)	–
Any intracranial hemorrhage	0.77 (0.43–1.39)	0.98 (0.45–2.09)	0.89 (0.71–1.11)	1.30 (0.47–3.61)	1.44 (1.00–2.09)	2.04 (0.55–7.63)
Symptomatic intracranial hemorrhage	1.29 (0.47–3.58)	0.52 (0.15–1.88)	0.93 (0.60–1.44)	1.06 (0.16–6.98)	2.35 (1.19–4.64)	–
Mortality	0.81 (0.40–1.67)	1.99 (0.55–2.09)	0.78 (0.64–0.96)	0.84 (0.27–2.61)	1.26 (0.85–1.87)	1.15 (0.20–6.64)

Adapted and cited from reference [47]

## Domestic development of tenecteplase: the T-FLAVOR

In Japan, no pharmaceutical company has handled tenecteplase, and it is not commercially available. The Boehringer Ingelheim, which holds European rights, has not acquired Japanese marketing rights. In 2018, the president of the Japan Stroke Society submitted a formal request for drug development to domestic pharmaceutical companies, but negotiations failed. As a result, no clinical data exist for tenecteplase use in Japan, even for myocardial infarction.

**Fig. 2:**
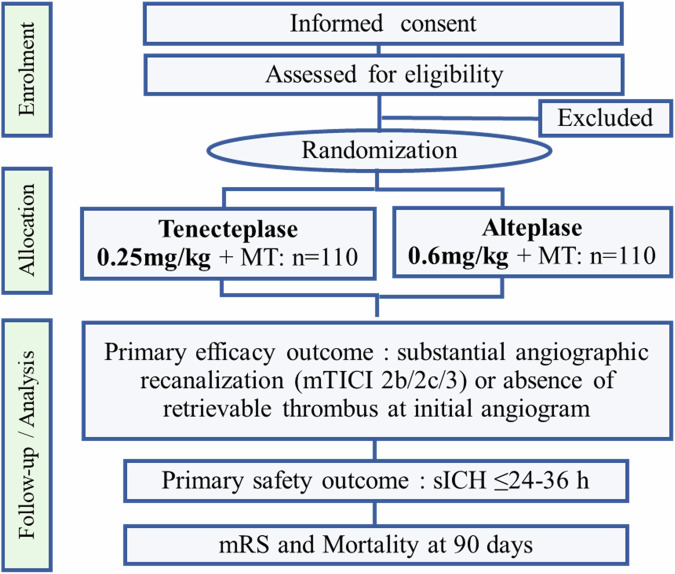
Assessment flow chart of the T-FLAVOR trial. mRS modified Rankin Scale, MT mechanical thrombectomy, sICH symptomatic intracranial hemorrhage

To overcome this situation, we planned an investigator-initiated, phase II, multicenter, prospective, randomized, open-label, masked-endpoint, superiority trial named the Tenecteplase versus alteplase For LArge Vessel Occlusion Recanalization (T-FLAVOR; Japan Registry of Clinical Trials [jRCTs] 051210055) [48]. This is the only trial using tenecteplase in Japan. Since the control group should receive officially approved treatment in Japan, the dose of alteplase was set at 0.6 mg/kg, official dose in Japan that is lower than globally approved dose. This is the world’s first trial to compare 0.25 mg/kg tenecteplase with 0.6 mg/kg alteplase. T-FLAVOR was conducted under the Category B Advanced Medical Treatment program authorized by the Japanese Ministry of Health, Labour and Welfare (MHLW) and funded by a governmental funding agency, Japan Agency for Medical Research and Development (AMED). The trial involves 18 institutions, centralized management at NCVC, and an imaging core lab at Iwate Medical University (Table 4). The safety confirmation phase began in 2021, and the randomized comparative phase started in 2022. The trial design of the randomized comparative phase was based on the EXTEND-IA TNK [29] and summarized in Table 5 and Fig. 2. We included adult patients presenting with acute ischemic stroke with occlusion of the major cerebral arteries who met eligibility for intravenous thrombolysis with alteplase and who can commence thrombolysis within 4.5 h of onset and mechanical thrombectomy within 6 h.

**Table 4:** Central and site investigators of T-FLAVOR

Central	
Principal Investigator	Kazunori Toyoda (National Cerebral and Cardiovascular Center [NCVC]), Teruyuki Hirano (Kyorin University)
Steering committee	Teruyuki Hirano (Kyorin University), Masatoshi Koga (NCVC), Nobuyuki Sakai (Kobe City Medical Center General Hospital), Kazunori Toyoda (NCVC)
Protocol committee	Hiroyuki Kawano (Kyorin University), Kanta Tanaka (NCVC), Katsuhiro Omae (NCVC), Koji Iwasaki (Osaka University)
Data monitoring	Mayumi Fukuda-Doi, Haruko Yamamoto (both for NCVC)
Statistician	Kenta Tanaka, Katsuhiro Omae (both for NCVC)
Independent safety monitoring board	Takehiko Nagao (Chair, Nippon Medical School Musashi Kosugi Hospital), Tetsuro Ago (Kyushu University), Makoto Nakajima (Kumamoto University), Shoji Sanada (Kobe University), Tomomi Yamada (Osaka University)
Central imaging reading board	Makoto Sasaki (Chair, Iwate Medical University), Ryo Itabashi (Iwate Medical University), Shoji Matsumoto (Fujita Health University), Hiroshi Yamagami (University of Tsukuba), Norio Yamashita (Iwate Medical University)
Central pharmacy	Naoki Hayakawa, Kazuyoshi Kawabata, Yoshiko Une (NCVC for all)
Trial Secretariat	Manabu Inoue, Tomohide Yoshie, Kanta Tanaka, Naruhiko Kamogawa, Yoshiko Hayashi (NCVC for all)
**Clinical sites**	**Site Investigators**
National Cerebral and Cardiovascular Center, Suita	Masatoshi Koga, Masafumi Ihara
Kobe City Medical Center General Hospital, Kobe	Nobuyuki Sakai, Tsuyoshi Ohta
Kyorin University, Mitaka	Teruyuki Hirano
Kurashiki Central Hospital, Kurashiki	Masaki Chin
Iwate Prefectural Central Hospital, Morioka	Naoto Kimura, Michiko Yokozawa
Nippon Medical School Hospital, Tokyo	Kazumi Kimura
Nagasaki University Hospital, Nagasaki	Akira Tsujino
Kumamoto Red Cross Hospital, Kumamoto	Tadashi Terasaki
Kokura Memorial Hospital, Kitakyushu	Taketo Hatano
National Hospital Organization Kyushu Medical Center, Fukuoka	Takahiro Kuwashiro
Hyogo Medical University, Nishinomiya	Shinichi Yoshimura
Tokai University Hospital, Isehara	Eiichiro Nagata
St. Marianna University Toyoko Hospital, Kawasaki	Toshihiro Ueda
Kyoto Second Red Cross Hospital, Kyoto	Yoshinari Nagakane
Saitama Medical University International Medical Center, Saitama	Shinichi Takahashi
Kagoshima City Hospital, Kagoshima	Fumio Miyashita
Saiseikai Fukuoka General Hospital, Fukuoka	Kazutaka Sonoda
St. Mary’s Hospital, Kurume	Kenji Fukuda

**Table 5:** Trial design of the randomized comparative phase of T-FLAVOR

Item	Details
Number of Participants	220
Target disease	Acute Ischemic Stroke
Study Type	Interventional
Study Design	Randomized, Two-arm, Open-label, Controlled
Main Inclusion Criteria	- Acute ischemic stroke - Age ≥ 20 years (no upper age limit) - Both sexes eligible- Eligible to receive intravenous thrombolysis based on recommendations of Japanese alteplase guideline - Eligible to commence intravenous thrombolysis within 4.5 h of onset - Occlusion of the internal carotid artery, middle cerebral artery (M1 or M2), or basilar artery confirmed by CT angiography or MR angiography - Eligible to commence mechanical thrombectomy (arterial puncture) within 6 h of onset - Written informed consent obtained from the patient or legal representative
Main Exclusion Criteria	- Pre-stroke disability (modified Rankin Scale ≥ 4) - More than 4.5 h since stroke onset or last known well time - Known allergy to contrast media - Terminal illness with expected life expectancy less than 1 year - Pregnant, breast-feeding, or of child-bearing potential - Currently participating in or planning to participate in another clinical trial during the study period - Judged as inappropriate for this study by the principal or sub-investigator
Intervention	Intravenous administration of tenecteplase (0.25 mg/kg) or alteplase (0.6 mg/kg), allocated 1:1
Primary Efficacy Endpoint	Substantial angiographic recanalization (mTICI 2b/2c/3) or absence of retrievable thrombus on the initial angiogram
Primary Safety Endpoints	- Symptomatic intracranial hemorrhage within 24–36 h after administration - All-cause mortality at 90 days
Secondary Endpoints	- ≥8 points reduction in the NIH Stroke Scale score or reaching 0–1 at 72 h after thrombolysis - Shift analysis of modified Rankin Scale (mRS) at 90 days - mRS 0–1 or unchanged from pre-stroke mRS at 90 days - mRS 0–2 or unchanged from pre-stroke mRS at 90 days
Discontinuation Criteria	- Occurrence of an adverse event requiring discontinuation as judged by the investigator - Withdrawal of consent by the participant - Serious bleeding complications making continuation difficult - Use of prohibited concomitant medications or therapies - Determined to be inappropriate as a study participant - Inability to conduct required assessments or examinations due to participant circumstances - Other reasons deemed appropriate for discontinuation by the investigator

Adapted and cited from reference [48]

Due to a lack of domestic supply, we imported tenecteplase from overseas. However, global demand, the COVID-19 pandemic, extreme yen depreciation, and the United States price hikes led to temporary shortages. We overcame this through crowdfunding and additional AMED support, completing enrollment of 220 patients in March 2025 and follow-up was completed in July 2025 Results are expected later 2025. The trial utilizes the Network for Clinical Stroke Trials system established with AMED support in 2015 [49].

The ultimate goal of T-FLAVOR is regulatory approval of tenecteplase in Japan for acute stroke. We have submitted the drug to the Unapproved Drugs Review Committee in the MHLW and are negotiating with potential manufacturers, with support from the Japan Stroke Society.

## How tenecteplase could transform acute stroke care

As shown by recent major RCTs, tenecteplase is at least as effective and safe as alteplase. It has shown superior efficacy in stroke due to large vessel occlusion and facilitates early recanalization, potentially avoiding mechanical thrombectomy. Compared to alteplase, which requires a 1-h infusion, tenecteplase is administered as a single bolus, making it particularly useful in urgent stroke care settings where rapid intervention is essential. It is ideal for rural or island areas relying on telemedicine and helicopter transport. Furthermore, tenecteplase is less expensive than alteplase in many countries. With rising global demand, there may be future reductions in alteplase production. Early adoption of tenecteplase in Japan would mitigate the impact of any alteplase shortage. Broader tenecteplase use could improve outcomes, increase thrombolysis rates, promote equitable care nationwide, reduce physician burden, and lower healthcare costs. Approval of tenecteplase at the international standard dose (0.25 mg/kg) could also increase opportunities for participation in global trials from Japan.

A major ethical issue common to all emergency trials, including this one, is the need to obtain informed consent in time-sensitive situations. Globally, deferred consent models are often used. Japan must seriously address this issue. The REFINED-IC project, funded by AMED, is working to establish appropriate consent procedures for hyperacute stroke trials in Japan by comparing international practices and regulations.

Following the approval of tenecteplase, there has been growing momentum worldwide for new stroke research utilizing tenecteplase. A representative example of this movement is A multi-faCtorial, mulTi-arm, multi-staGe randomized, gLOBal Adaptive pLatform trial for stroke (ACT-GLOBAL) [50]. This is a platform clinical trial that enrolls adult patients aged 18 and older with acute ischemic stroke without exclusion criteria and conducts multiple parallel studies to address various clinical questions surrounding intravenous thrombolysis with tenecteplase. Researchers from many countries, including Japan, have expressed their intention to participate. Until now, the primary focus of research has been on comparative trials between tenecteplase and alteplase aimed at gaining approval for tenecteplase. However, going forward, it will be essential to concentrate on studies that explore the optimal use of tenecteplase in stroke patients under various conditions. Many clinical questions, such as tenecteplase use for patients on direct oral anticoagulants and the combination of tenecteplase with neuroprotective agents, which were not covered in past development trials, are now being considered as research topics. In particular, the comparison between the internationally standard dose of 0.25 mg/kg and a lower dose of 0.18 mg/kg of tenecteplase as a core trial from ACT-GLOBAL is an especially noteworthy candidate topic for Japanese researchers, who have long used low-dose alteplase in clinical practice.

In addition, the Japanese guidelines for the use of alteplase include a few unique recommendations that differ from global ones, and it is necessary to verify through domestic trials whether similar recommendations can be applied to tenecteplase. For example, in Japan, the eligibility criteria for alteplase administration in patients developing stroke while taking direct oral anticoagulants are considerably relaxed compared with global standards [51, 52]; it is therefore important to determine whether a similar relaxation of criteria can be justified for tenecteplase.

Given Japan’s 9-year delay in approving alteplase, we must not repeat history. With broad support, we aim to secure rapid approval for tenecteplase and ensure timely and effective stroke care across Japan.
